# Spontaneous Coronary Artery Dissection and Fibromuscular Dysplasia: A Case Series and Genetic Links

**DOI:** 10.7759/cureus.54105

**Published:** 2024-02-13

**Authors:** Quang Le, Amit Bhandari, Julie Fleischer, Avinash Murthy

**Affiliations:** 1 Hospital Medicine, University of Missouri School of Medicine, Columbia, USA; 2 Internal Medicine, St. John's Hospital, Springfield, USA; 3 Pediatrics, Southern Illinois University School of Medicine, Springfield, USA; 4 Cardiology, Prairie Heart Institute, Springfield, USA

**Keywords:** fibromuscular disease, genetic screening, coronary fibromuscular dysplasia, coronary artery disease, sponateonus coronary artery dissection

## Abstract

Spontaneous coronary artery dissection (SCAD) is a rare cause of myocardial infarction in young women. An association of fibromuscular dysplasia (FMD) with SCAD has been well established; a significant proportion of SCAD patients may have typical FMD findings in other noncoronary arteries. The current consensus recommends arterial imaging screening from head to pelvis using computed tomography angiography (CTA) or magnetic resonance angiography (MRA) in SCAD. Genetic testing for FMD should be considered in high-risk cases. We present two cases of SCAD associated with FMD and discuss the significance of genetic screening in such patients.

## Introduction

Spontaneous coronary artery dissection (SCAD) is a non-atherosclerotic cause of myocardial infarction (MI), predominantly in young and middle-aged women, accounting for up to 4% of acute coronary syndrome (ACS) cases overall and 35% in females under 50. Most well-known etiologies involve an independent or overlapped spectrum of fibromuscular dysplasia (FMD), connective tissue diseases (CTDs), pregnancy, and genetic susceptibility [1]. In a previous cohort study, FMD was found in approximately 25% to 86% of SCAD cases [2]. We present a case series of SCAD with underlying fibromuscular dysplasia. We discuss the importance of screening for fibromuscular dysplasia in SCAD and genetic counseling and screening in patients with high-risk features.

## Case presentation

Case 1

**Figure 1:**
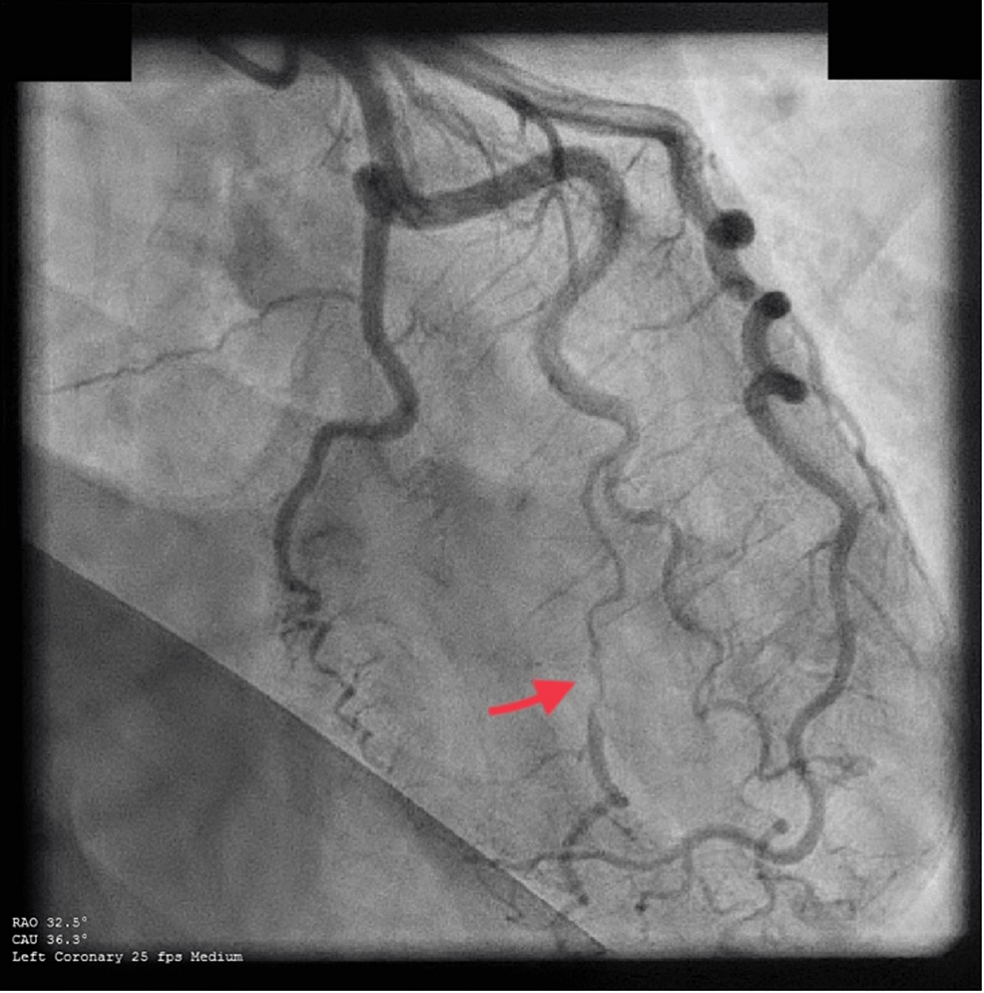
SCAD of the first obtuse marginal artery. Proximal and mid-portion narrowing of the first obtuse marginal artery with normal caliber at the distal segment and TIMI 3 flow, typical of type 2A based on Yip-Saw classification. SCAD: Spontaneous coronary artery dissection, TIMI: Thrombolysis in myocardial infarction.

A 50-year-old female, a non-smoker, arrived at the emergency room with chest pain and dizziness. Her family history was significant for abdominal aortic aneurysms (AAA) requiring repair in his father in his 60s and sudden due to AAA in her paternal first cousin in his 40s. Additionally, her paternal uncle and maternal grandfather had AAA, though the exact ages were unspecified. Electrocardiography (ECG) showed a normal sinus rhythm without new ST-T changes. High-sensitivity Troponin I levels were elevated and progressively increasing. Chest computed tomography angiography (CTA) ruled out pulmonary embolism, while echocardiography (ECHO) revealed normal left ventricular function but identified a dilated ascending aorta of 3.31 cm. Urgent coronary angiography showed several centimeters of proximal and mid-portion narrowing of the lower branch of the first obtuse marginal artery with the distant portion returning to the normal caliber of a TIMI-3 flow (Figure 1), consistent with a diagnosis of SCAD.

Further imaging with CTA of the head/neck and abdomen displayed characteristics indicative of fibromuscular dysplasia. Genetic testing of a 92-gene panel for connective tissue disorders and smooth muscle aortopathy yielded normal results. The patient declined whole exome sequencing (WES) testing. Despite aortic dilation and a strong family history of AAA, she did not exhibit clinical features of CTDs. After receiving conservative management involving medication (metoprolol tartrate, ranolazine, clopidogrel, aspirin, and a high-intensity statin), the patient was discharged with a favorable prognosis. 

Case 2

**Figure 2:**
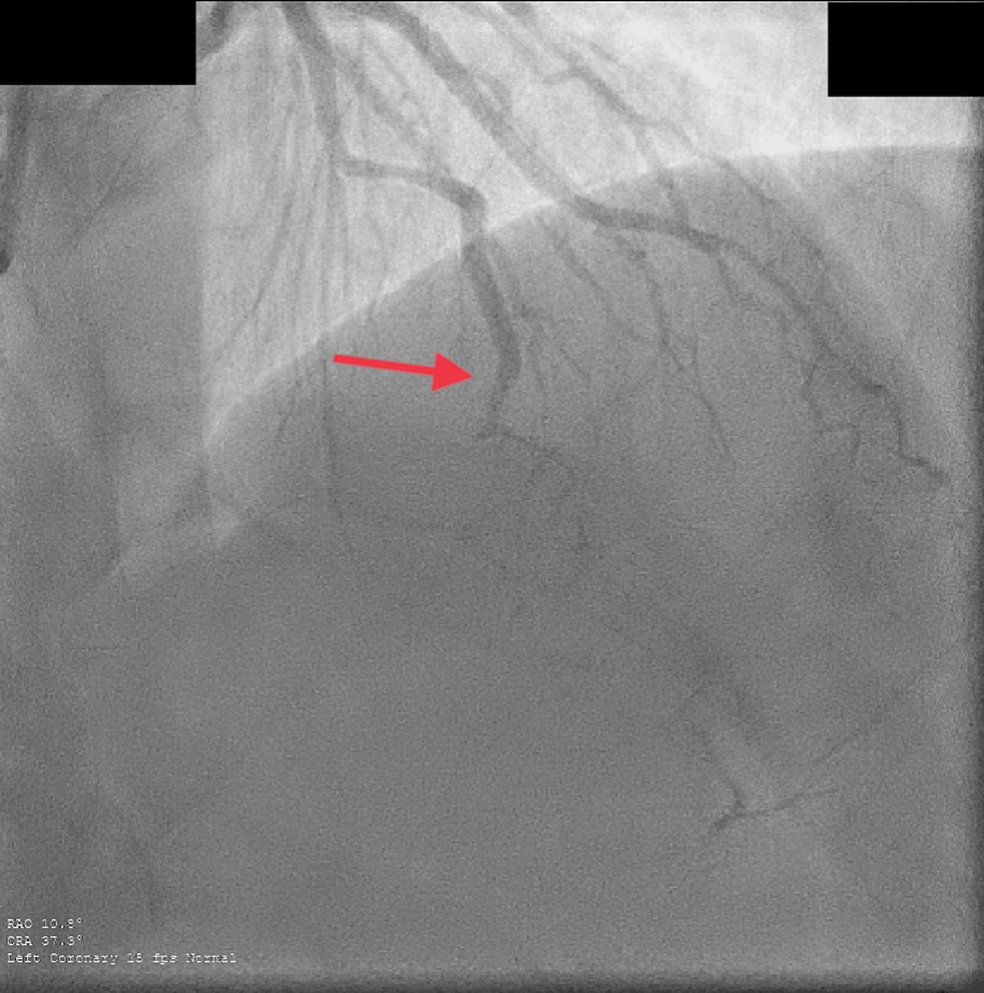
SCAD of mid-LAD artery. Complete stenosis of mid-LAD with collateral vessels supplying the apex, classified as type 2B Yip-Saw system. SCAD: Spontaneous coronary artery dissection, LAD:  Left anterior descending artery.

A 43-year-old female presented to the emergency room with chest pain. Her electrocardiogram (EKG) showed ST elevation in the lateral leads. Troponin levels were elevated to 13155 ng/L. Coronary angiography revealed a 50% stenosis in the mid-vessel of the left anterior descending artery (LAD) and a 100% blockage in the mid-vessel of the first diagonal branch. A drug-eluting stent was placed. The patient had a previous ACS event two years earlier, with similar findings in the LAD and first diagonal artery. She was on dual antiplatelet therapy (aspirin and Brilinta) but switched to aspirin and prasugrel after the stent placement. Three weeks later, she returned to the ER with chest pain, and troponin levels were elevated again. Repeat angiography showed complete stenosis in the mid-LAD with collateral vessels supplying the apex (Figure 2), consistent with SCAD. 

FMD was highly suspected based on head and neck CTA (Table 1). The patient had additional clinical features like a high-arched palate, joint hypermobility, and a Beighton score of 4/9, suggestive of an underlying genetic condition. Genetic testing of a 92-gene panel for connective tissue disorders and smooth muscle aortopathy was normal. She declined whole-exome sequencing (WES).

**Table 1:** A summary of the patient’s other relevant features. CTA: Computed tomography angiography, FMD: Fibromuscular dysplasia, ICA: Internal carotid artery. SD: Standard deviation, SMA: Superior mesenteric artery.

	Case 1	Case 2	
FMD artery lesions	Right renal artery -bilateral iliac arteries -bilateral cervical internal carotid arteries V2 -bilateral vertebral arteries V3 of right vertebral artery	Right internal carotid artery -left vertebral artery	
CTA head/neck	Aneurysmal dilation of the distal cervical right ICA with minimal beaded appearance	Distal right ICA tortuous with slight luminal irregularity -left vertebral artery with slight irregularity	
CTA chest -abdomen -pelvis	Saccular aneurysm of the SMA	Not present	
Dilation of ascending aorta	3.31 cm	Not present	
Short stature	Not present	Present (<2SD)	
Small feet	Not present	Present (<3SD)	
Short metatarsal	Not present	Present	
Brachydactyly	Not present	Present	
Beighton score	1/9	4/9	
Palate	Not abnormality	High arched	
Relevant history	None	Graves’s disease, vitiligo, carpal tunnel syndrome	

## Discussion

SCAD occurs when a weakened arterial wall leads to the formation of an intramural hematoma, resulting in compression of the true lumen and ischemic events [1,2]. SCAD is classified into three types using the Yip-Saw angiographic system. Type 1 exhibits contrast staining in the arterial wall with multiple radiolucent lumens. Type 2 demonstrates a significant abrupt change in arterial caliber, causing diffuse narrowing of more than 20 mm. Type 2 is further divided into 2A, with dissection bordered by normal proximal and distal arterial segments, and 2B, with dissection extending to the distal tip. Type 3 manifests as focal or tubular stenosis resembling atherosclerosis. Type 2 lesions are subtle and often overlooked during coronary angiography, while type 3 lesions are most challenging to differentiate from atherosclerosis and may require optical coherence tomography or intravascular ultrasound for accurate diagnosis [2,3].

SCAD has a diverse range of etiologies with increasing evidence of genetic predisposition. Common causes include FMD, connective tissue disease, systemic inflammatory diseases, and pregnancy [1]. The clinical presentation of SCAD can mimic ACS [4]. Triggers for SCAD can include physical or emotional stress, medications, and hormonal changes [1]. Clinical examination, ECG, and cardiac biomarkers are used to assess myocardial injury. While coronary angiography is the gold standard, SCAD's diffuse and subtle nature may not always provide a definitive diagnosis. Additional imaging modalities like intravascular ultrasound (IVUS) and optical coherence tomography (OCT) can characterize the dissection anatomy and guide treatment decisions [4].

SCAD management includes several approaches: medical therapy, revascularization, and coronary artery bypass grafting (CABG). Unlike atherosclerotic myocardial infarction, percutaneous coronary intervention (PCI) in SCAD carries a higher risk of complications, making a conservative approach preferable for symptom relief and close monitoring. Non-revascularized SCAD cases typically heal within 30 days. PCI may be used in unstable patients or those who fail conservative therapy, while CABG is reserved for failed PCI or high-risk anatomy such as left main or proximal 2-vessel dissection [1,4]. Due to limited clinical trials, there are no clear-cut guidelines on the medical management of SCAD. Medications may target left ventricular dysfunction (beta-blockers, ACE inhibitors, ARBs) or alleviate chest pain (nitrates, calcium channel blockers, ranolazine). Routine statin use is not recommended unless indicated for primary prevention of atherosclerotic diseases [1,4]. In a single-center study by Feldbaum et al., beta blockers and aspirin were the most prescribed medications for SCAD patients [5]. Standard dual antiplatelet therapy (DAPT) is used in patients who receive stents during PCI. However, the optimal duration is unclear, and the risk of bleeding must be weighed against the potential benefit of preventing recurrent events. A recommended approach is 2-4 weeks of DAPT followed by low-dose aspirin for 3 to 12 months [1]. The long-term prognosis for SCAD is generally favorable; most patients experience complete or near-complete resolution. However, recurrence and adverse events like heart failure, arrhythmias, and sudden cardiac arrest remain concerns, especially in patients with underlying conditions like FMD or CTDs [4].

FMD is a non-inflammatory vascular disorder leading to arterial stenosis, occlusion, aneurysm, or dissection. It primarily affects women, with a ratio of 9:1 compared to men. While FMD can occur in any arterial bed, it most commonly involves the renal, extracranial carotid, and vertebral arteries (in approximately 65% of cases) [6]. SCAD may be the most common presentation of FMD involving coronary arteries. The characteristic "string-of-beads" feature rarely occurs in coronaries. Type 2 SCAD lesions are the most typical, while non-atherosclerotic focal stenosis, dissection, or arterial tortuosity are less common [4]. The prevalence of FMD can be up to 45% and is considered a predictor of future major adverse cardiac events in SCAD patients [3,7]. The current consensus recommends comprehensive arterial imaging from head to pelvis using CTA or magnetic resonance angiography (MRA) in SCAD patients. However, a study done by Feldbaum et al. in a tertiary care center in the US showed that less than 20% of patients with SCAD underwent comprehensive screening. The authors suggested a better screening strategy for extra-coronary vascular abnormalities by a multidisciplinary approach, which utilizes SCAD registries, online SCAD support groups, and protocol development to complete imaging of multiple vascular beds at one visit [5].

Genetic testing for SCAD and FMD is gaining interest as newer technologies emerge. A study by Wang et al. focused on high-risk SCAD patients (peripartum patients, recurrent cases, or individuals with a family history of arteriopathy) found that 1 out of 5 individuals in this subgroup had a rare genetic variant such as COL3A1, Loeys-Dietz syndrome genes, ADAMTSL4, and LRP1 [8]. The finding suggests the idea of genetic screening in these specific patient groups. The American Heart Association (AHA) currently does not recommend routine genetic testing in all patients with SCAD. Instead, it should be tailored for cases with certain characteristics, such as a strong family history, association with other vascular disorders (aortic aneurysms or dissections), multiple arterial lesions, or young age of onset [6]. While commercial gene panels offer genes associated with CTDs and smooth muscle-related conditions, several genes related to SCAD and FMD such as PHACTR1, LRP1, LIMA1, ATP2B1, and PTGIR are not currently included in these panels. Additionally, genes like OBSCN, DYNC2H1, PKD1, PKD2, and RNF213 are available on commercial panels but not specifically for aneurysms or arteriopathies. Therefore, whole exome or whole genome sequencing would be a more comprehensive approach. However, they can often be very expensive. It may be beneficial to develop new gene panels that specifically target patients with SCAD or FMD, covering the possible genetic causes that overlap between these conditions (Table 2). Such panels would allow for more targeted and cost-effective genetic testing in these patient populations. 

**Table 2:** A summary of genes currently being utilized for SCAD and FMD. [8-10] * Indicates genes being tested on the 92 gene panel for connective tissue disorders used for two of our case series. CTD: Connective tissue disease, EDS: Ehlers-Danlos syndrome, FMD: Fibromuscular dysplasia, SCAD: Spontaneous coronary artery dissection, TAA: Thoracic aortic aneurysm.

Gene	SCAD	FMD	TAA	Other CTDs	Protein type	Related panel
PHACTR1	Present	Present	-	None	Phosphatase and actin regulator	None
PTGIR	Present	Not present	-	None	Receptor for prostacyclin	None
LRP1	Present	Present	-	None	Low-density lipoprotein receptor	None
LIMA1	-	Present	-	None	Actin filament depolymerization inhibitor	None
ATP2B1	-	Present	-	None	Intracellular calcium homeostasis	None
FBN1*	-	-	Marfan syndrome	None	Connective tissue	None
COL4A1*	Present	-	Present	Present	Connective tissue	None
COL3A1*	Present	-	Vascular EDS	Present	Connective tissue	None
ALDH18A1*	Present	-	Present	Present	Cutis laxa	None
ACVR1*	Present	-	Present	None	Fibro-dysplasia ossificans progressiva	None
PRKG1*	Present	Present	Present	None	Smooth muscle	None
MYLK*	-	Present	Present	None	Smooth muscle	None
OBSCN	-	Present	-	None	Sarcomere signaling proteins	Arrhythmia cardiomyopathy
DYNC2H1	-	Present	-	None	Dynein-2 complex of cilia	Tumor panel
RNF213	-	Present	-	None	Moyamoya disease	Tumor panel
COL5A1	-	Present	-	None	Connective tissue	None
PKD1/2	Present	-	-	None	Polycystic kidney disease	None

## Conclusions

There is strong evidence to support FMD as the most common associated condition with SCAD. Screening for FMD is recommended for SCAD patients. Genetic testing should be considered in high-risk SCAD or cases with strong family history or other vascular implications. A multidisciplinary approach is essential for the diagnosis and management of patients with SCAD with FMD, incorporating a careful clinical history and physical examination, electrocardiography, biomarker testing, imaging studies, and advanced diagnostic testing.
